# Computer-Assisted Morphometric Comparative Analysis of Argyrophilic Nucleolar Organizer Regions (AgNORs) in Leukoplakia With Dysplasia and Oral Squamous Cell Carcinoma

**DOI:** 10.7759/cureus.54471

**Published:** 2024-02-19

**Authors:** Roshin C Nararyanan, Bastian T Sebastian, Suhana H Sulaikha, Cimmy Augustine, Teenu Thomas, Anjali Sudhakaran

**Affiliations:** 1 Oral Pathology and Microbiology, Mahe Institute of Dental Sciences & Hospital, Mahe, IND; 2 Prosthodontics, Mahe Institute of Dental Sciences & Hospital, Mahe, IND; 3 Public Health Dentistry, Mahe Institute of Dental Sciences & Hospital, Mahe, IND; 4 Oral and Maxillofacial Surgery, Mahe Institute of Dental Sciences & Hospital, Mahe, IND

**Keywords:** profile area per nucleus, nuclear profile area, dysplasia, morphometric analysis, oscc, oral squamous cell carcinoma, leukoplakia, nucleolar organizer region, silver nitrate, agnor

## Abstract

Background

Oral squamous cell carcinoma (OSCC) and oral leukoplakia (OL) with dysplasia are closely linked conditions in the oral cavity, with the latter often indicating precancerous changes, underscoring the urgency of early detection and intervention. Histopathological confirmation is crucial for accurate diagnosis. The nucleolar organizer region (NOR), specifically analyzed through silver-staining (argyrophilic NORs), provides insights into nuclear changes associated with the lesion. Computer-assisted morphometric analysis enhances precision and objectivity in evaluating AgNOR-related parameters.

Aim

To conduct a computer-assisted morphometric comparison of AgNORs using various NOR-related parameters in cases of OSCC and leukoplakia with dysplasia and to evaluate their diagnostic significance.

Materials and methods

A computer-assisted morphometric analysis was conducted using various NOR-related parameters, such as nuclear profile area, single AgNOR profile area per nucleus, total AgNOR profile area per nucleus, and number of AgNOR profiles per nucleus on a total sample of 90 specimens, which includes leukoplakia with dysplasia (30), OSCC (30), and a control group, including 30 samples of normal oral mucosa. A comparison was conducted on the morphometric values between the groups under investigation. Tukey's multiple comparison tests and ANOVA were used to analyze the data and determine the differences between the groups.

Results

The present investigation revealed a significant difference in all four AgNOR-related parameters between leukoplakia and OSCC in comparison to the control group (normal oral mucosa). Comparing OL (41.78 ± 0.46) and OSCC (62.78 ± 0.47) to the control group (35.93 ± 0.99), the mean value of nuclear profile area (A Nuc) was significantly greater. In comparison to the control group (3.40 ± 0.09), the mean value of a single AgNOR profile area per nucleus (A NOR) was found to be relatively lower in both research groups, OL (2.00 ± 0.02) and OSCC (1.39 ± 0.01). The total AgNOR profile area per nucleus (TA NOR) had a mean value of 10.61 ± 0.69 in OL and 12.05 ± 0.28 in OSCC, respectively, compared to 7.82 ± 0.38 in the control group. The study found that there was more number of profiles of AgNORs per nucleus (n NOR) in the study groups of OL (5.30 ± 0.29) and OSCC (8.69 ± 0.19) than in the control group (2.32 ± 0.11).

Conclusion

The parameters linked to the NOR are biologically informative and easy to check regularly in a pathology lab. Additionally, AgNORs give us important information that enables us to study the range of nuclear changes in malignant and potentially malignant lesions.

## Introduction

Oral squamous cell carcinoma (OSCC) is the most prevalent form of malignancy in the mouth, making up 90% of all malignancies in the mouth [1]. It is India's third-most common cancer and a major public health concern [2]. The majority, if not all, cases of OSCCs are preceded by clinically apparent but frequently asymptomatic alterations of the oral mucosa. These modifications frequently exhibit a largely white composition. These whitish lesions, known as oral leukoplakia (OLs), have the potential to become malignant. They are predominantly white and cannot be wiped off, and they occur on the oral mucosa. The approximate prevalence of OL is around 0.1%, although it may differ throughout various regions of the world. OL primarily manifests in individuals aged 30-40 years and is significantly more prevalent among smokers compared to non-smokers. The potential direct role of alcohol is less evident in the development of oral cancer compared to other factors [3].

The nucleolus, the largest subnuclear component of the cell, is formed around areas of chromosomes that include sequences of tandem repetitive ribosomal RNA (rRNA) genes known as nucleolar organizer regions (NORs). These ribosomal DNA loops are argyrophilic and are hence referred to as argyrophilic nucleolar organizer regions (AgNORs). AgNORs serve as indicators of nucleolar activity and replication. The morphometric approach, specifically employing computer-assisted area measuring, will be used to assess AgNORs [4].

The investigation was done on the clinical importance of AgNOR number and area in developing lesions from OL to OSCC. The study examined various parameters pertaining to the quantity and dimensions of AgNORs in tissue samples of the healthy oral mucosa, leukoplakia, and OSCC. Understanding these parameters can provide insights into tumour behaviour and serve as an adjunct diagnostic method.

This study focused on quantitatively and qualitatively analyzing AgNORs in oral tissue samples to investigate their clinical significance in the progression of lesions by examining the potential for proliferation and tissue stability. The study aims to contribute to understanding OSCC and its precursor lesions, potentially enhancing the timely identification and treatment of oral cancer.

## Materials and methods

Design

This study adopts a retrospective observational design to analyze the significance of AgNOR count and area in different study groups, namely, cases with OL (with dysplasia) and oral squamous cell carcinoma, along with a control group consisting of normal oral mucosa samples.

Ethical considerations

Ethical approval for conducting this study was obtained from the Institutional Ethical Committee with reference number MINDS/PG-ETHICAL/03/2016-17.

Inclusion and exclusion criteria

The inclusion criteria are as follows: histologically confirmed cases of OL (with dysplasia) and oral squamous cell carcinoma, available clinical details and sufficient tissue for analysis, and normal oral mucosal tissue samples for the control group. The exclusion criteria are as follows: cases without adequate clinical details or sufficient tissue for analysis and tissues exhibiting processing artifacts.

Sample size derivation

A total of 60 cases of histologically diagnosed OSCC and OL (30 OL and 30 OSCC) were considered for the study, along with 30 sections of normal oral mucosal tissue for the control group. The sample size was determined based on the availability of archival data meeting the inclusion criteria within the study period of 2016-2019.

Variables studied

The primary variables studied include nuclear profile area, single AgNOR profile area per nucleus, total AgNOR profile area per nucleus, and the number of profiles of AgNOR per nucleus in the study groups and the control group.

Technique

By using a soft tissue semi-automatic rotary microtome (Leica RM2245), the paraffin-embedded tissue blocks were sectioned (two sections from each block were sectioned into 5 μm thickness). Then, the staining of one section was done with hematoxylin and eosin (H&E), and the other section was stained with silver stain preparation of the AgNOR solution (modified technique for AgNORs staining, as suggested by Smith et al.) [5]. The histopathological slides were viewed using the Trinocular Research Microscope Olympus BX-53 (Olympus America Scientific Equipment Group, Center Valley, PA) with a digital camera. Morphometric analysis was done using the image analysis software ProgRes®capturePro 2.8.8 (Jenoptik, Jena, Germany). Two independent observers conducted the analysis to minimize subjectivity.

Morphometric analysis

Histological grading of the lesions with hematoxylin and eosin sections was followed by morphometric analysis of the corresponding silver-stained sections. To minimize subjectivity, two observers independently evaluated all the readings.

**Figure 1:**
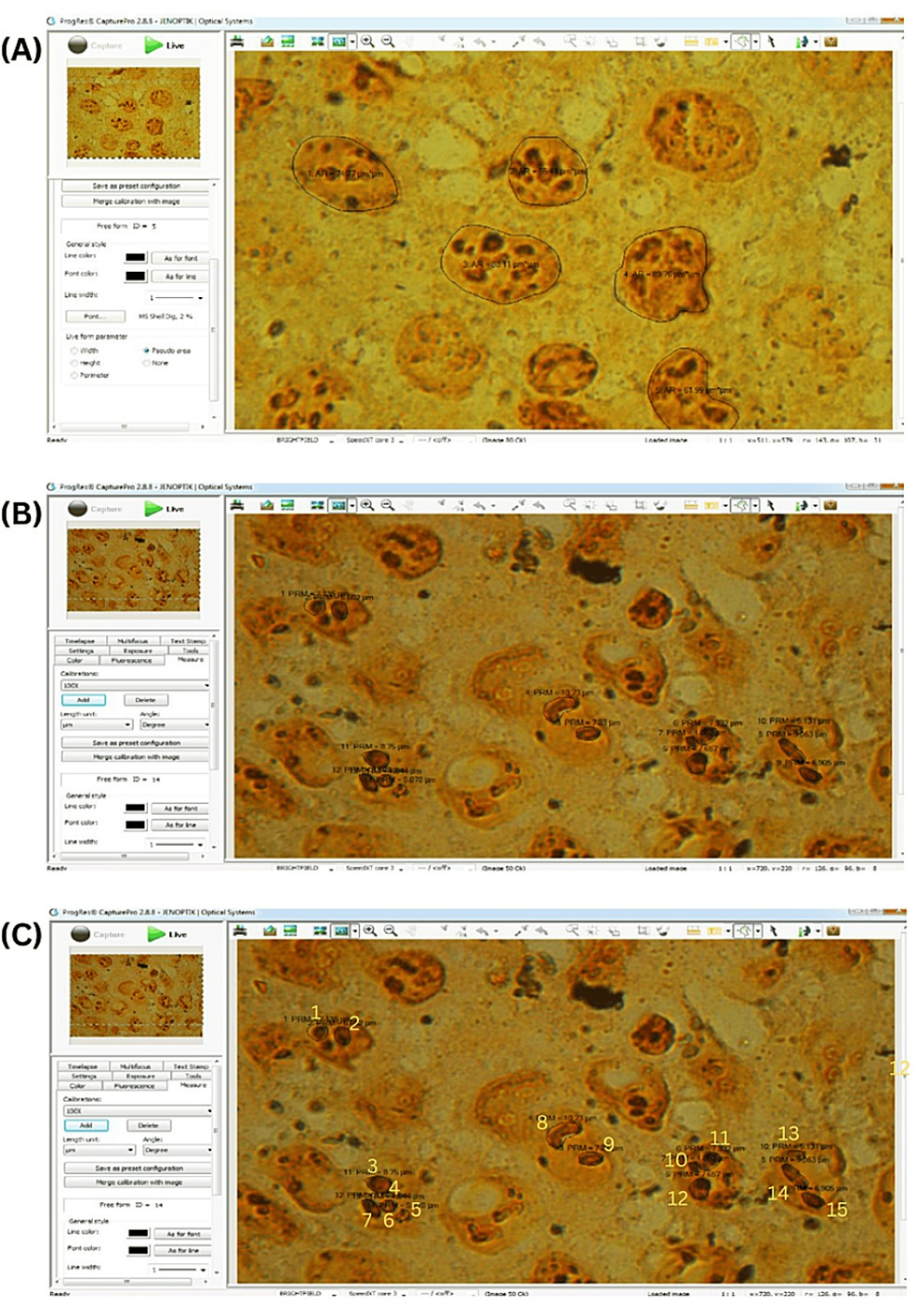
Photomicrographs of the morphometric parameters evaluated using the Windows-based image analysis software (ProgRes® CapturePro 2.8.8). A: Photomicrograph demonstrating measurement of nuclear profile area (A Nuc). B: Photomicrograph demonstrating measurement of single AgNOR profile area (A NOR) and total AgNOR profile area (TA NOR). C: Photomicrograph demonstrating the number of AgNOR (n NOR).

For nuclear and AgNOR morphometric analysis, images were captured using a trinocular research microscope (Olympus BX-53) with a digital camera. Windows-based image analysis software (ProgRes®capturePro 2.8.8) was used for accurate measurements, and magnification calibration was performed using a stage micrometre before measurements. The final images on the monitor were at a magnification of 1000x, and these images were saved for the research (Figure 1).

The measurements were performed using the tools for measurement available in the image analyzer system, with five microscopic fields selected randomly from each section. The selection process began with the first representative field on the left and proceeded to include five fields from each area in the following field. The stage readings were recorded for reevaluation [5].

The mouse pointer was used to trace the five largest nuclei with clear borders in each chosen field. In each nucleus, the number of silver-stained, black, or brownish-black dots (AgNORs) was counted. AgNOR dots that were clustered together were considered as a single AgNOR dot and tallied as such. The software performed an automated calculation of the nuclear area.

**Figure 2:**
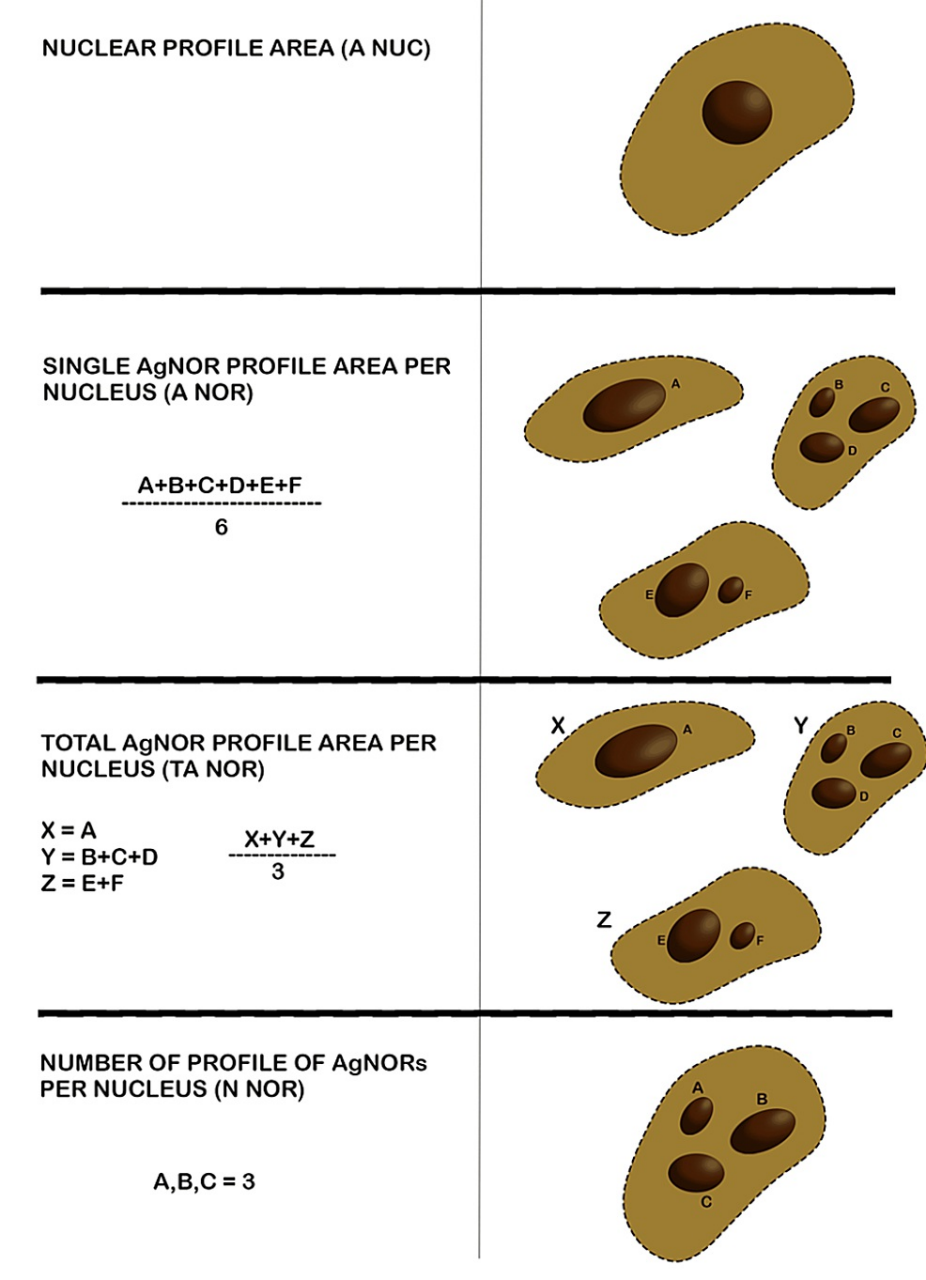
Schematic representation of the nucleolar organizer region-related morphometric parameters evaluated.

By using advanced software, the research precisely measured the nuclear profile area (A Nuc), single AgNOR profile area per nucleus (A NOR), total AgNOR profile area per nucleus (TA NOR), and the number of profiles of AgNORs per nucleus (N NOR) (Figure 2).

A Nuc: The nuclear area was quantified in square micrometers. The nuclear perimeter was traced to get measurements, and the software automatically computed the nuclear area. Five nuclei with clear boundaries were chosen from five different fields.

A NOR: The single AgNOR profile area was quantified in square microns. The AgNOR perimeter was traced for measurement, and the software automatically calculated the AgNOR area.

Here, the area of the total number of AgNORs per nucleus was calculated using morphometric analysis, and the areas of all AgNORs of 25 nuclei of five different fields were summed and then divided by the total number of AgNORs of all 25 nuclei.

TA NOR: The unit of measurement for the AgNOR profile area per nucleus was square microns. After tracing the AgNOR perimeter for measurement, the AgNOR area was automatically determined by the software. Morphometric analysis is used to determine the overall AgNOR area per nucleus. The area of all 25 nuclei's AgNOR was summed, and the result was divided by 25.

N NOR: The number of AgNOR profiles per nucleus (N NOR) was recorded. In all cases, 25 nuclei were picked in the same manner, as previously described. The number of distinct and solitary dots within each nucleus was measured, and the mean value was computed for each example. In cases when two or more dots were densely clustered within a nucleus, rendering it impossible to accurately determine the exact count of individual dots within the cluster, the entire cluster was considered as a single entity during the counting process.

Statistical analysis

The descriptive statistics, including the mean and standard deviation, were computed. Tukey multiple comparison tests and one-way ANOVA were used to compare the groups. The software programs utilized in this study were Microsoft Excel and Statistical Product and Service Solutions (SPSS, version 22.0; IBM SPSS Statistics for Windows, Armonk, NY) for Windows to do statistical analysis. The p-values less than 0.05 were considered statistically significant.

## Results

**Figure 3:**
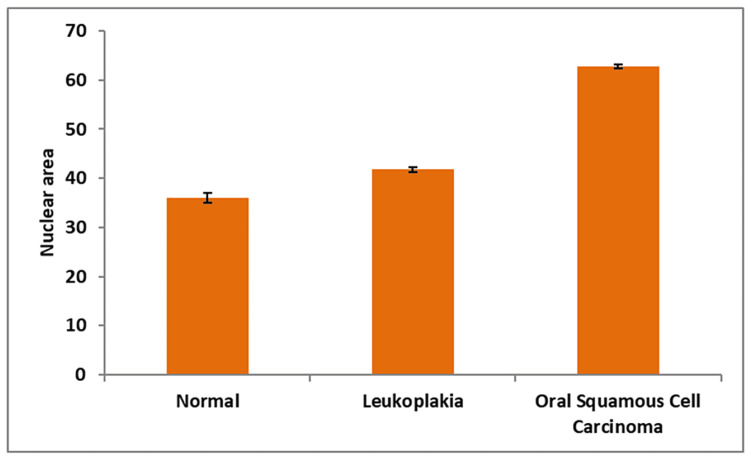
Mean value of the nuclear profile area (A Nuc) between three groups.

**Figure 4:**
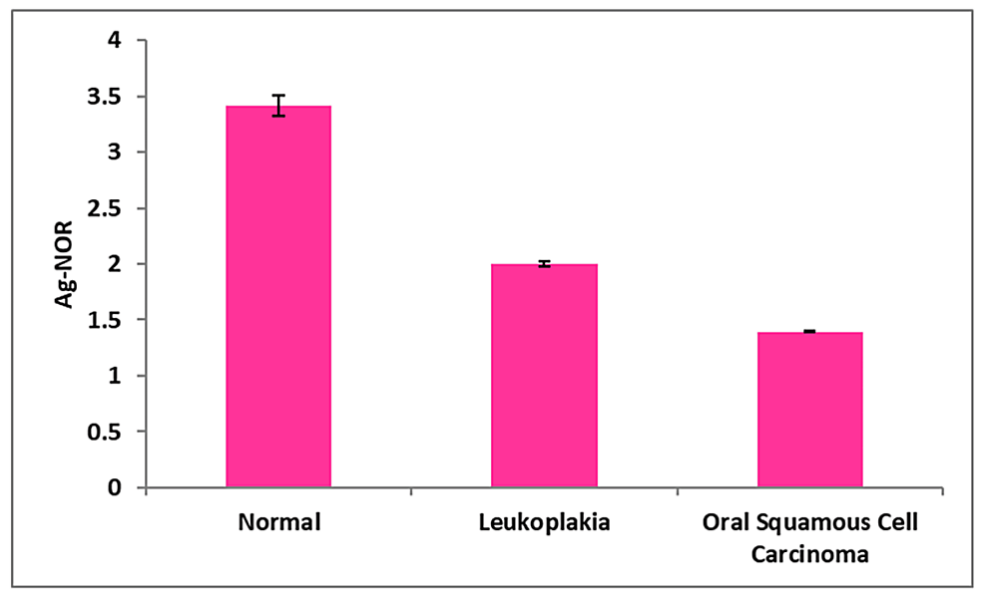
Mean value of a single AgNOR profile area per nucleus (A NOR) between three groups.

**Figure 5:**
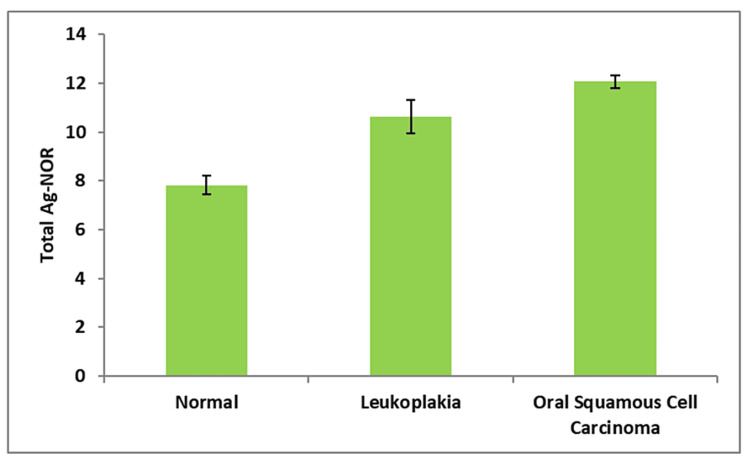
Mean value of the total AgNOR profile area per nucleus (TA NOR) between three groups.

**Figure 6:**
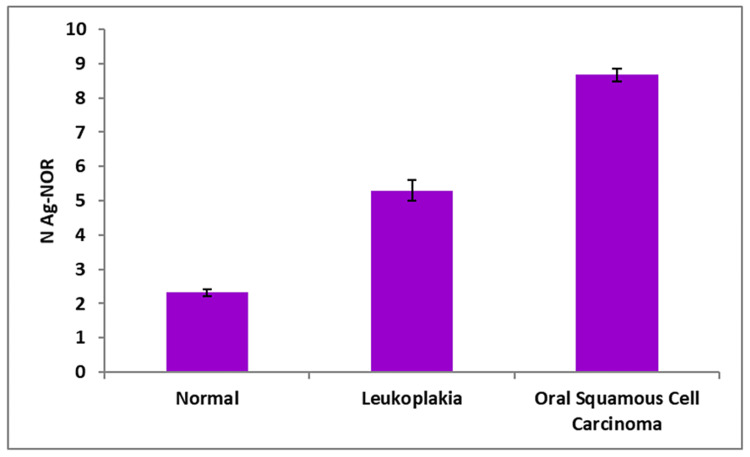
Mean number of profiles of AgNORs per nucleus (n NOR) between three groups.

In comparison to the control group (normal oral mucosa), all four AgNOR-related parameters of leukoplakia and oral squamous cell carcinoma differed significantly in the present study (Table 1). In contrast to the control group, which had a mean nuclear profile area (A Nuc) of 35.93 ± 0.99, cases of oral leukoplakia (41.78 ± 0.46) and oral squamous cell carcinoma (62.78 ± 0.47) exhibited substantially greater A Nuc values (Figure 3). The mean AgNOR profile area per nucleus (A NOR) was lower in oral leukoplakia (2.00 ± 0.02) and oral squamous cell carcinoma (1.39 ± 0.01) compared to the control group (3.40 ± 0.09) (Figure 4). The mean total AgNOR profile area per nucleus (TA NOR) was higher in oral leukoplakia (10.61 ± 0.69) and oral squamous cell carcinoma (12.05 ± 0.28) compared to the control group (7.82 ± 0.38 ) (Figure 5). The number of profiles of AgNORs per nucleus (n NOR) was significantly larger in both research groups: 5.30 ± 0.29 for oral leukoplakia and 8.69 ± 0.19 for oral squamous cell carcinoma, compared to the control group, which had an average of 2.32 ± 0.11 profiles (Figure 6).

**Table 1:** Comparison of morphometric parameters between the study groups.

Group	Description	N	A Nuc (μm^2^)	A NOR (μm^2^)	TA NOR (μm^2^)	n NOR
Control Group	Normal	30	35.93 ± 0.99 (34.22-37.67)	3.40 ± 0.09 (3.27-3.57)	7.82 ± 0.38 (7.08-8.34)	2.32 ± 0.11 (2.08-2.60)
Study Group	Leukoplakia	30	41.78 ± 0.46 (41.11-43.05)	2.00 ± 0.02 (1.96-2.03)	10.61 ± 0.69 (9.33-11.61)	5.30 ± 0.29 (4.72-5.72)
	Oral Squamous Cell Carcinoma	30	62.78 ± 0.47 (61.95-63.66)	1.39 ± 0.01 (1.38-1.40)	12.05 ± 0.28 (11.74-12.48)	8.69 ± 0.19 (8.40-8.92)
ANOVA (f-value)			12,668.248	10,604.603	594.194	6652.504
p-value			<0.001	<0.001	<0.001	<0.001

## Discussion

Squamous cell carcinoma ranks as the world's sixth most frequently diagnosed malignancy within the craniofacial and cervical areas, encompassing the oral, laryngeal, and pharyngeal areas. It constitutes 90% of all oral malignancies and 40% of malignancies that affect the head and neck and is the prevailing neoplasm affecting the oral cavity [6,7].

It is well-accepted that OSCC is practically preceded by observable changes in the oral mucosa. These alterations typically manifest as white patches (leukoplakia) or red patches (erythroplakia). Detecting and closely monitoring these potentially cancerous lesions and conditions allows healthcare practitioners to identify and manage oral cancer in its early intraepithelial stages, such as epithelial dysplasia. Often, these stages occur before the onset of invasive OSCC of the oral cavity. The timely identification and management of these potentially malignant lesions play a crucial role in enhancing the prognosis and results for individuals diagnosed with oral cancer. By promptly addressing these potentially malignant lesions and conditions, healthcare professionals can significantly improve the prognosis and outcomes for patients affected by oral cancer [8,9].

While hematoxylin and eosin staining is commonly employed for the histological diagnosis of carcinomas, there are cases where the histopathological characteristics may not offer adequate clarity in determining the precise nature of these tumors. Alternative methods, such as the silver colloid method for staining NORs inside the nucleus, have shown to be more useful histochemical techniques that offer further insights into the cellular situation [10].

Numerous research investigations have showcased the potential utility of argyrophilic AgNORs as markers for diagnosing different types of tumors. AgNOR counts can distinguish OSCC from the normal epithelium. In addition, a number of studies imply that AgNORs may serve as distinguishing markers for mild and moderate epithelial dysplasia [11]. The AgNOR approach, with its low cost and easily reproducible results, offers great potential, particularly in resource-constrained environments.

While there have been previous reports on the morphometric analysis of normal oral epithelium and mucosal diseases, such as leukoplakia [11,12], lichen planus [13], squamous cell papilloma [14], and squamous cell carcinoma [15,16], there is a scarcity of studies specifically examining nuclear and AgNOR morphometry in relation to OL and various grades of OSCC.

The objective of our research was to assess and analyze the morphological alterations in the A Nuc, single A NOR, TA NOR, and n NOR across normal oral mucosa, OL, and OSCC. A comparison was made between OL and OSCC of the oral cavity as well.

Many researchers have discussed the histology of OL and OSCC using mean AgNOR numbers and their importance. However, few studies have been conducted utilizing multiple AgNOR metrics, such as A Nuc, single A NOR, TA NOR, and n NOR. Assessing cellular proliferation in histological material is crucial for conventional histopathology, and many methods can quantify it. Ideally, procedures should be simple, reproducible, and appropriate to normal histological and cytological preparations [17,18].

In the present study, the nuclear profile area from the section of normal oral mucosa of 30 individuals showed a mean value of 35.93 ± 0.99 µm^2^. This finding was consistent with the prior research conducted by Cabrini et al. [14], Thippeswamy et al. [19], and Singh et al. [17]. Cabrini et al. [14] studied sections from 10 normal oral mucosa, and the mean nuclear profile area of normal oral mucosa showed a value of 44.88 ± 9.03 µm^2^ [14]. Thippeswamy et al. [19] studied sections from 10 normal oral mucosa, and the mean nuclear profile area of normal oral mucosa showed a value of 41.42 ± 1.37 µm^2 ^[19]. Singh et al. [17] studied sections from 10 normal oral mucosa, and the mean nuclear profile area of normal oral mucosa showed a value of 36.19 ± 7.83 µm^2 ^[17].

In this study, the nuclear profile area from the sections of 30 cases of OL demonstrated a mean value of 41.78 ± 0.46 µm^2^. This result was in accordance with the prior research done by Shabana et al. [20] and Singh et al. [17]. Shabana et al. [20], in their research of 20 OL, observed a mean nuclear profile area of 33.13 ± 3.30 µm^2^. Singh et al. [17], in their study of 20 OL, observed a mean nuclear profile area of 41.97 ± 6.43 µm^2^.

In our study, the nuclear profile area for oral squamous cell carcinoma showed mean values of 62.78 ± 0.47 µm^2^. This finding was consistent with the prior research conducted by Cabrini et al. [14], Schwint et al. [21], and Singh et al. [17]. In a study conducted by Cabrini et al. [14], they examined six cases of moderately differentiated OSCC. They found that the mean nuclear profile area was reported to be 66.61 ± 23.44 µm^2 ^[14]. In a study conducted by Schwint et al. [21], the authors studied 12 OSCC and observed a mean nuclear profile area of 68.05 ± 24.22 µm^2 ^[21]. Singh et al. [17], in their study of 20 OSCC, observed a mean nuclear profile area of 62.36 ± 26.63 µm^2 ^[17].

A substantial difference was observed in the mean nuclear profile area among the normal oral mucosa, OL, and OSCC. The observed result entails an increase in the nuclear profile area throughout the process of malignant transformation in epithelial cells. The findings of the present study match with prior research in this field.

In this study, the single AgNOR profile area per nucleus of normal oral mucosa displayed a mean value of 3.40 ± 0.09 µm^2^. This finding was consistent with the prior research conducted by Cabrini et al. [14], Spolidorio et al. [22], Thippeswamy et al. [19], and Singh et al. [17]. Cabrini et al. [14] conducted a study on 10 cases and found that the single AgNOR profile area per nucleus in normal oral mucosa was 3.32 ± 2.01 µm^2 ^[14]. In 12 cases of normal oral mucosa, Spolidorio et al. [22] found that the single AgNOR profile area per nucleus was 3.17 ± 0.57 µm^2 ^[22]. Thippeswamy et al. [19] in their study of 10 cases showed that the single AgNOR profile area per nucleus in normal oral mucosa was 3.02 ± 0.17 µm^2^ [19]. Singh et al. [17] studied sections from 10 normal oral mucosa, and the single AgNOR profile area per nucleus of normal oral mucosa showed a value of 3.45 ± 0.37 µm^2^.

In this study, the single AgNOR profile area per nucleus of OL displayed a mean value of 2.00 ± 0.02 µm^2^. This finding aligns with the earlier studies undertaken by Muzio et al. [23], Spolidorio et al. [22], and Singh et al. [17]. In a study conducted by Muzio et al. [22], they examined 21 cases of OL to determine the mean value of a single AgNOR profile area per nucleus. Among the 21 cases, 13 exhibited a low degree of dysplasia, four exhibited a moderate degree of dysplasia, and four exhibited a severe degree of dysplasia. The mean value of the single AgNOR profile area per nucleus in the 13 cases with a low degree of dysplasia was found to be 2.99 ± 1.36 µm^2^. In the four cases with a moderate degree of dysplasia, the mean value was 5.01 ± 1.04 µm^2^, while in the four cases with a severe degree of dysplasia, the mean value was 4.03 ± 0.85 µm^2 ^[23]. In their investigation of 23 cases of leukoplakia, Spolidorio et al. [22] found that the mean value of the single AgNOR profile area was 4.98 ± 0.77 µm^2^. In a study conducted by Singh et al. [17], sections from 20 cases of leukoplakia were examined. The researchers found that the single AgNOR profile area per nucleus was 2.76 ± 0.41 µm^2 ^[17].

In our investigation, the mean of the single AgNOR profile area per nucleus in cases of OSCC was determined to be 1.39 ± 0.01 µm^2^. These results were in accordance with similar studies undertaken by Cabrini et al. [14], Schwint et al. [21], and Singh et al. [17]. Cabrini et al. [14] studied six cases of OSCC in the oral cavity (moderately differentiated) and reported a mean single nuclear profile area per nucleus of 1.38 ± 0.52 µm^2^. Schwint et al. [21] studied 12 OSCC and observed a mean single nuclear profile area per nucleus of 1.65 ± 0.61 µm^2^. In a study of sections from 20 patients of OSCC, Singh et al. [17] found that the mean single AgNOR profile area per nucleus was 1.61 ± 0.30 µm^2^.

Our study results showed a decrease in the mean single AgNOR profile area per nucleus in OSCC when compared to the normal epithelium and OL of the oral cavity. The decrease in size of a single NOR could be due to a spectrum of nuclear alterations occurring within the epithelium as the lesion progresses from normal oral mucosa to OL and further to OSCC. As mentioned by Sano et al. [24], it would be a manifestation of abnormal proliferation and differentiation processes associated with the production of new oncogenic proteins found in carcinomas.

In this research, the TA NOR of normal oral mucosa displayed a mean value of 7.82 ± 0.38 µm^2^. This finding was consistent with the prior research conducted by Schwint et al. [21], Spolidorio et al. [22], Thippeswamy et al. [19], and Singh et al. [17]. In a study conducted by Schwint et al. [21], the mean total AgNOR profile area per nucleus in normal oral mucosa was found to be 6.78 ± 1.87 µm^2^ based on a sample size of 10 cases [21]. Similarly, Spolidorio et al. [22] reported a mean value of 4.28 ± 0.97 µm^2^ for the total AgNOR profile area per nucleus in normal oral mucosa, with a sample size of 12 cases [22]. Thippeswamy et al. [19] conducted a study whereby they analyzed a total of 12 patients in order to assess the mean value of the total AgNOR profile area per nucleus in the normal oral mucosa. The findings revealed that the mean value was 7.88 ± 0.27 µm^2 ^[19]. Singh et al. [17] studied sections from 10 normal oral mucosa, and the total AgNOR profile area per nucleus of the normal oral mucosa displayed a value of 5.27 ± 0.67 µm^2 ^[17].

In our research, the TA NOR of OL had a mean value of 10.61 ± 0.69 μm^2^. This finding was consistent with the prior research conducted by Muzio et al. [23], Spolidorio et al. [22], and Singh et al. [17]. In a study conducted by Muzio et al. [23], a total of 21 cases of OL were examined. The researchers found that the average value of the total AgNOR profile area per nucleus was 5.28 ± 1.218 µm^2^ in 13 cases of leukoplakia with a low degree of dysplasia. Additionally, the mean value was 7.113 ± 1.095 µm^2^ in four moderate leukoplakia cases and 8.123 ± 0.462 µm^2^ in four severe cases. Spolidorio et al. [22] in their study of 23 cases of OL displayed that the mean value of the total AgNOR profile area per nucleus was 7.13 ± 1.58 µm^2^. Singh et al. [17] in their study of 20 cases of OL showed that the mean value of the total AgNOR profile area per nucleus was 6.81 ± 1.14 µm^2^.

In our study, the total AgNOR profile area per nucleus of OSCC showed a mean value of 12.05 ± 0.28 μm^2^. These results were inconsistent with the prior research conducted by Cabrini et al. [14], Schwint et al. [21], and Singh et al. [17] Cabrini et al. [14] studied six OSCC (moderately differentiated) cases and reported a mean total AgNOR profile area per nucleus of 10.80 ± 4.22 µm^2^. Schwint et al. [21] in their study of 12 cases of OSCC showed a mean total AgNOR profile area per nucleus of 9.97 ± 2.96 µm^2^. Singh et al. [17] studied sections from 20 cases of OSCC and showed a mean total AgNOR profile area per nucleus of 11.30 ± 2.43 µm^2^.

These results suggest that the total AgNOR profile area per nucleus is seen increasing as the lesion advances from OL to OSCC when compared to the normal mucosa of the oral cavity, and the results obtained in our study were in accordance with the previous studies. The number of AgNORs per nucleus significantly increased when the cells displayed abnormal characteristics.

The current study observed that the n NOR of normal oral mucosa had a mean value of 2.32 ± 0.11. These results were inconsistent with the prior research conducted by Cabrini et al. [14], Usta et al. [25], Arora et al. [26], Thippeswamy et al. [19], Machado et al. [27], Lingegowda et al. [18], and Singh et al. [17]. In their study of 10 normal oral mucosa, Cabrini et al. [14] found that the mean n NOR was 2.95 ± 1.42. Usta et al. [25] in their study among 40 normal oral mucosa showed that the n NOR of normal oral mucosa had a mean value of 3.5 ± 0.3. Arora et al. [26] in their study among normal oral mucosa showed that the n NOR of normal oral mucosa had a mean value of 2.6 ± 0.21 [26]. Thippeswamy et al. [19] in their study among 10 normal oral mucosa showed that the n NOR of normal oral mucosa had a mean value of 2.71 ± 0.10 [19]. Machado et al. [27] in their study among normal oral mucosa (exfoliative cytology) showed that the n NOR of normal oral mucosa had a mean value of 2.93 ± 0.21. In their study among normal oral mucosa, Lingegowda et al. [18] showed that the n NOR of normal oral mucosa had a mean value of 2.49 ± 0.30. Singh et al. [17] studied sections from 10 normal oral mucosa, and the n NOR of normal oral mucosa had a mean value of 2.58 ± 0.13.

The number of profiles of n NOR of OL in our study showed a mean value of 5.30 ± 0.29. This result could be compared with the previous research performed by Warnakulasuriya et al. [28], who in their study of 23 cases showed that the number of profiles of n NOR in 15 cases of epithelial dysplasia had a mean value of 5.61 ± 4.63 [28]. Arora et al. [26] in their study of 15 cases of OL showed that the n NOR had a mean value of 3.40 ± 0.58. Lingegowda et al. [18], in their study among mild, moderate, and severe grades of epithelial dysplasia, showed that the n NOR had a mean value of 3.15 ± 0.11, 3.43 ± 0.28, and 4.82 ± 0.18, respectively. Singh et al. [17] studied sections from 20 cases of OL, and the n NOR had a mean value of 5.68 ± 0.40.

The n NOR of OSCC in our study had a mean value of 8.69 ± 0.19. These results were inconsistent with the prior research conducted by Cabrini et al. [14], Warnakulasuriya et al. [28], Schwint et al. [21], Gulia et al., Girish et al. [15], and Singh et al. [17]. In their study of six cases of semi-differentiated OSCC, Cabrini et al. [14] observed that the mean n NOR was 8.04 ± 2.44. In a study of 11 cases of OSCC, Warnakulasuriya et al. [28] found that the mean n NOR was 8.37 ± 6.11. Schwint et al. [21], in their study of 12 cases of OSCC, showed that the n NOR had a mean value of 6.56 ± 2.33. Gulia et al. [29], in their study of OSCC, showed that the n NOR had a mean value of 4.71 ± 0.81, 6.34 ± 0.89, and 8.70 ± 0.65 in well-differentiated, moderately differentiated, and poorly differentiated OSCC, respectively. Girish et al. [15], in their study among different grades of OSCC, showed that the n NOR had a mean value of 6.59 ± 0.20, 7.96 ± 0.22, and 8.84 ± 0.24 in well-differentiated, moderately differentiated, and poorly differentiated OSCC, respectively [15]. Singh et al. [17], in their study of 20 cases of OSCC, showed that the n NOR had a mean value of 7.92 ± 0.70.

The findings of this investigation suggest that there was a greater mean AgNOR count observed in cases of OSCC in comparison to normal oral mucosa. These results align with the findings reported in prior studies stated before.

This implies that, when epithelial cells undergo malignant transformation, the number of AgNORs in their nuclei increases. The preceding findings indicate that the mean AgNOR count could be used as a valuable tool for assessing the prognosis of dysplastic lesions and cases of OSCC [17].

The AgNOR technique is effective for studying premalignant and malignant lesions, but it has limitations such as difficulty in interpreting results and background staining, making weakly stained NORs harder to identify and resolve. Inadequate filtering can cause staining granules in the cytoplasm, black precipitates on slides, and section fading within days.

One notable finding from the morphometric analysis is that the A Nuc, TA NOR, and n NOR showed a gradual increase from normal oral mucosa to OL and further to OSCC. Meanwhile, the single A NOR showed a decrease in value from normal oral mucosa to OL and further to OSCC. Hence, our research findings underscore the importance of AgNOR as a quantitative and discriminative tool among the biological parameters associated with premalignant or early malignant changes. This proves to be an easy method that can be monitored in a routine pathology laboratory and aids in the detection of initial cellular changes.

## Conclusions

The study shows that an increase in the A Nuc is seen when epithelial cells go through dysplastic alterations and malignant transformation. Increased TA NOR and n NOR in malignancies signify the level of malignancy or the activation of NORs. Changes in proliferation and differentiation mechanisms are shown by a decrease in the mean single A NOR. AgNORs are more irregular, smaller, and more prevalent in oral squamous cell carcinomas. When identifying oral cavity lesions and assessing cell proliferative activity, AgNOR evaluation can be a helpful tool. Future research should focus on the AgNOR contour index for individual cases to identify early epithelial changes and for timely intervention to prevent the disease from advancing to a clearly malignant state.
